# Disorders of Digestion

**Published:** 1886-12-25

**Authors:** 


### Disorders of Digestion.*

First Notice.—Introductory.

"Socrates," it has been said, "brought Philosophy from the clouds. The English have degraded her to the kitchen." A truer account of the matter would be this: "When Philosophy came down from the clouds, she visited the Greeks, who first admired and then made a toy of her. Whereupon she left them. In later times, when she came to the English, they also admired, but finding her a gentle instructress, they asked her to show them the *reason* which underlay every natural process, *and every practical art*. This was exactly what she desired, and so she took up her abode with them, promising to remain as long as she found them in so docile and reasonable a temper." For many centuries Philosophy treated Medicine with scorn, but that was only because Medicine, being superstitious and stupid, asked no aid from her. In recent times, Medicine, having learnt better, has gone to school to Philosophy, with results which are familiar to all men. Dr. Lauder Brunton is one of those who seeks a reason for every rhyme. And though to seek is not always to find all we wish to know, yet it is always to find something, and the more thorough and intelligent the search, the richer is the reward which is ultimately discovered.

It is strange, and indeed almost inexplicable, that whilst the mind has absorbed the study of the learned for thousands of years, the body should have been deemed of little or no account. The discovery of the circulation of the blood dates no further back than the Commonwealth—little more than two hundred years. The theories of the circulation, of the digestion, of disease in all its forms, were such as to excite the amused scepticism of the vulgar and the contempt of the learned. In the glorious reign of Elizabeth, when Bacon and Raleigh, Burleigh and Shakespeare, not to mention lesser stars in that brilliant literary and political firmament, were at their meridian—many of the theories of disease, and the remedies prescribed for them, were so far removed from fact and reason, so inherently absurd, indeed, that one can hardly believe it possible that they were ever really believed in even by those who maintained them most strenuously, and most largely profited by them. Now we have changed all that. It is not our purpose to sing a pæan in praise of modern as against ancient times, but it is our wish to convince men that, whatever faults modern medicine may be charged with, ignorance, stupidity, and intellectual laziness are not among them. What the medical mind can do, that the medical mind has long been striving to do. "Prejudice," as Tennyson commands, has been "cut against the grain." "The old" has been "rung out," and "the new rung in" with a completeness which could hardly have been exceeded. Dr. Lauder Brunton's book is entirely modern. It would have been no disadvantage to the general reader if a brief glance had been thrown upon the past, in order that an instructive contrast with the present might have been seen. We shall return to the book again for the purposes of a more detailed review, which cannot but be instructive, and practically important to all who are conscious of the possession of a digestive apparatus. In the meantime, it may be commended, both to professional and general readers, as a thoroughly intelligent, complete, and practically useful treatise, on the highly important questions of which it treats. The drawings and diagrams are well done, the printing is excellent, and the book in every way worthy of the subject, and of the distinguished reputation of its author.

* *Disorders of Digestion.* By T. Lauder Brunton, M.D., F.R.S. London: Macmillan and Co.

